# Adenylate Kinase Isozyme 3 Regulates Mitochondrial Energy Metabolism and Knockout Alters HeLa Cell Metabolism

**DOI:** 10.3390/ijms23084316

**Published:** 2022-04-13

**Authors:** Koichi Fujisawa, Maina Wakazaki, Aya Matsuzaki, Toshihiko Matsumoto, Naoki Yamamoto, Takafumi Noma, Taro Takami

**Affiliations:** 1 Department of Gastroenterology and Hepatology, Yamaguchi University Graduate School of Medicine, Minami Kogushi 1-1-1, Ube Yamaguchi 755-8505, Japan; mshiyzm_1022@yahoo.co.jp (M.W.); smy_hos@yahoo.co.jp (A.M.); tm0831@yamaguchi-u.ac.jp (T.M.); t-takami@yamaguchi-u.ac.jp (T.T.); 2 Department of Environmental Oncology, Institute of Industrial Ecological Sciences, University of Occupational and Environmental Health, 1-1 Iseigaoka, Yahatanishi-ku, Kitakyushu 807-8555, Japan; 3 Yamaguchi University Health Administration Center, 1677-1 Yoshida, Yamaguchi 753-8511, Japan; nao-yama@yamaguchi-u.ac.jp; 4 Department of Nutrition and Health Promotion, Hiroshima Jogakuin University, 4-13-1, Ushitahigashi, Higashiku, Hiroshima 732-0063, Japan; tkdntaka@gmail.com

**Keywords:** adenylate kinase 3, nucleotide metabolism, phosphoenolpyruvate, GTP metabolism

## Abstract

The balance between oxidative phosphorylation and glycolysis is important for cancer cell growth and survival, and changes in energy metabolism are an emerging therapeutic target. Adenylate kinase (AK) regulates adenine nucleotide metabolism, maintaining intracellular nucleotide metabolic homeostasis. In this study, we focused on AK3, the isozyme localized in the mitochondrial matrix that reversibly mediates the following reaction: Mg^2+^ GTP + AMP ⇌ Mg^2+^ GDP + ADP. Additionally, we analyzed AK3-knockout (KO) HeLa cells, which showed reduced proliferation and were detected at an increased number in the G1 phase. A metabolomic analysis showed decreased ATP; increased glycolytic metabolites such as glucose 6 phosphate (G6P), fructose 6 phosphate (F6P), and phosphoenolpyruvate (PEP); and decreased levels of tricarboxylic acid (TCA) cycle metabolites in AK3KO cells. An intracellular ATP evaluation of AK3KO HeLa cells transfected with ATeam plasmid, an ATP sensor, showed decreased whole cell levels. Levels of mitochondrial DNA (mtDNA), a complementary response to mitochondrial failure, were increased in AK3KO HeLa cells. Oxidative stress levels increased with changes in gene expression, evidenced as an increase in related enzymes such as superoxide dismutase 2 (SOD2) and SOD3. Phosphoenolpyruvate carboxykinase 2 (PCK2) expression and PEP levels increased, whereas PCK2 inhibition affected AK3KO HeLa cells more than wild-type (WT) cells. Therefore, we concluded that increased PCK2 expression may be complementary to increased GDP, which was found to be deficient through AK3KO. This study demonstrated the importance of AK3 in mitochondrial matrix energy metabolism.

## 1. Introduction

In cancer cells, the Warburg effect is known to suppress mitochondrial oxidative phosphorylation, changing it to glycolytically shifted metabolism, and alterations in energy metabolism have recently attracted attention as a therapeutic target [1]. Changes in the energy metabolism in cancer cells lead to the production of ATP through mitochondrial glycolysis and oxidative phosphorylation, and ATP needs to be transported to the necessary intracellular location. In addition to simple diffusion, the adenylate kinase (AK) and creatine kinase (CK) systems are known to transport ATP to where it is highly consumed in cells [2,3], but the details of the mechanisms mediating the metabolism have not been elucidated.

AK, an enzyme that regulates the metabolism of adenine nucleotides, is widely expressed in higher organisms and bacteria and catalyzes the ATP + AMP ⇌ 2 ADP reaction. AK maintains the homeostasis of intracellular nucleotide metabolism and is necessary in vivo for normal proliferation, differentiation, motility, and metabolic functions [4,5]. This enzyme has been reported to exist across many living organisms ranging from bacteria to animals. Furthermore, nine isozymes have been identified to date, including the cytoplasmic enzymes AK1, AK5, AK7, and AK8 [6,7,8], alongside the nuclear enzyme AK6 [9], AK2 in the mitochondrial intermembrane space, and AK3 and AK4 being localized in the mitochondrial matrix [10]. 

Prokaryotic cells such as bacteria and eukaryotic yeasts have only one type of AK enzymes and cannot survive without its activity. Furthermore, AK1 deficiency has been reported to cause hematological abnormality, whereas AK2 gene deficiency causes reticular dysgenesis and sensorineural deafness, which indicates the important roles of AK in differentiation [11,12]. Additionally, AK4 has been suggested to be involved in oxidative stress, and is reported to be one of the proteins that are increased following the administration of four types of hepatotoxic drugs such as carbon tetrachloride. In addition, AK4 has attracted increasing attention as the most important gene associated with mitochondrial ATP production [13]. To date, continued studies of AK have reported its intracellular and tissue-specific expression [14] and we reported that knocking down AK4 increases mitochondrial activity [15]. 

Interestingly, AK3, which exists in the mitochondrial matrix along with AK4, may be closely associated with metabolism in the mitochondrial matrix [16]. AK3 is thought to function in reversibly mediating the Mg^2+^ GTP + AMP ⇌ Mg^2^ + GDP + ADP reaction in the mitochondrial matrix. AK3 generates GDP and ADP using GTP produced by phosphorylation in the citric acid cycle at substrate level and AMP that exists in the matrix, respectively, and the generated GDP is utilized in the next cycle of the citric acid cycle, while the ADP is utilized as a substrate of mitochondrial ATP synthetase [17,18,19]. Moreover, AK3 has been reported to sensitize cancer cells that have acquired cisplatin-resistance by exposure to condensed tobacco vapor [20]. Furthermore, pancreatic Langerhans islet β-cells have low AK3 expression and, therefore, would not consume the GTP required for insulin secretion [21], but the function of AK3 is yet to be clarified. Although AK3 is reported to be homeostatically expressed in a variety of tissues, much remains unknown, especially regarding its relationship to GTP metabolism. Therefore, in this study, we evaluated the role of AK3 in energy metabolism in cancer cells by creating and analyzing an AK3 knock-out (AK3KO) HeLa cell line derived from cervical cancer cells.

## 2. Materials and Methods

### 2.1. Production of AK3KO HeLa Cells

The HeLa cells used to generate the AK3KO HeLa cells were purchased from the Japanese Collection of Research Bioresources and the process was conducted using a CRISPR-Cas9 system (Sigma-Aldrich, Tokyo, Japan) containing gRNA (gRNA target sequence: GTAGTGATGCGCGACGACACGG (2nd exon) and a Cas9 expression unit for AK3 and GFP in a single vector was used. Cells in which Sigma CRISPR is introduced and double-strand breaks are caused by Cas9 are efficiently obtained from cells exhibiting transient luminescence by GFP. Therefore, single cell-sorting was conducted using strongly GFP-positive cells with the MoFlo Astrios cell sorter (Beckman Coulter, Tokyo, Japan). The cells grown from a single cell were evaluated the expression of AK3 protein with Western blotting.

### 2.2. Proliferation Assay

A 96-well plate with 3000 bone marrow-derived mesenchymal stem cells (BMSCs) per well were seeded in different concentrations of deferoxamine (DFO), and cell proliferation was determined by measuring the area of the cells using the Incucyte HD imaging system (Essen BioScience, Ann Arbor, MI, USA). DFO was purchased from Novartis Pharma (Tokyo, Japan).

### 2.3. Western Blotting

In a sample buffer containing 62.5 mM Tris-HCl (pH6.8), 4% sodium dodecyl sulfate (SDS), 200 mM dithiothreitol, 10% glycerol, and 0.001% bromophenol blue at a weight ratio of 1:10 (*w*/*v*), cell pellets were homogenized to obtain a protein lysate, which was then boiled. The antibody products used were β-actin (Sigma-Aldrich, Tokyo, Japan), AK3, PCK2, SOD2 and SOD3 (Abcam, Tokyo, Japan).

### 2.4. Measurement of ATP

ATeam1.03 [22] was purchased from addgene (http://www.addgene.org, accessed on 24 December 2018, a gift from Dr. Takeharu Nagai) and was transfected into the cells, which were imaged using a fluorescence microscope. CFP and YFP fluorescence (475 and 527 nm, respectively) were evaluated for ATP non-binding and ATP-binding, respectively. The images were pseudocolored for the intensity ratio of YFP/CFP.

### 2.5. Total RNA Isolation

Total RNA was isolated from each sample using TRIzol reagent (Life Technologies, Tokyo, Japan) and purified using SV total RNA isolation system (Promega, Tokyo, Japan) according to the manufacturer’s instructions. RNA samples were quantified using an ND-1000 spectrophotometer (NanoDrop Technologies, Wilmington, DE, USA) and the RNA quality was confirmed using the Experion system (Bio-Rad Laboratories, Hercules, CA, USA).

### 2.6. SAGE

The Ion Ampliseq Transcriptome Human Gene Expression kit (Life Technologies, Tokyo, Japan) was used for library creation [23]. An ion proton next-generation sequencer library of the analysis beads was created, and an Ion PI IC 200 kit (Life Technologies, Tokyo, Japan) and Ion PI Chip kit v2 BC were used for sequencing with an Ion Proton next-generation sequencer. Genes with adjusted *p* < 0.05 and maximum expression >50 were identified as statistically significant. The results of the SAGE were integrated using IPA.

### 2.7. Metabolomic Analysis

A metabolomic analysis was performed according to the analysis company (Human Metabolome Technology Inc., Tsuruoka, Japan). Briefly, the culture medium was aspirated from a 10 cm cell culture dish and the cells were washed twice with 5% mannitol solution (10 mL first and then 2 mL). The cells were then treated with 800 µL methanol and left to stand for 30 s to inactivate the enzymes. Next, the cell extract was treated with 550 µL Milli-Q water containing internal standards (H3304-1002, Human Metabolome Technologies, Inc., Tsuruoka, Japan) and were allowed to incubate for another 30 s. 

The extract was collected, centrifuged at 2300× *g* and 4 °C for 5 min, and then 800 µL of the upper aqueous layer was centrifugally filtered through a Millipore 5-kDa cutoff filter at 9100× *g* and 4 °C for 120 min to remove the proteins. The filtrate was concentrated using a centrifuge and resuspended in 50 µL Milli-Q water for a capillary electrophoresis–mass spectrometry (CE-MS) analysis. To identify the affected metabolic pathways, a proof-of-knowledge-based IPA (Ingenuity Pathway Systems, Redwood City, CA, USA) was performed.

### 2.8. Statistical Analysis

The data are presented as the means ± standard deviation, with the significance level established at *p* < 0.05. Differences between the means of measurements were evaluated using an unpaired Student’s *t*-test (two-tailed). Differences in the means of more than two groups were assessed as follows. Normality tests were conducted for all groups with a significance level of 0.05, and dispersion tests were performed using Bartlett’s test. If all groups presented a normal distribution and equal variances, the Tukey–Kramer parametric method was used. In all other cases, the Steel–Dwass nonparametric method was used.

## 3. Results

### 3.1. AK3KO Decreases Proliferation and Number of Cells in G2 Phase

**Figure 1:**
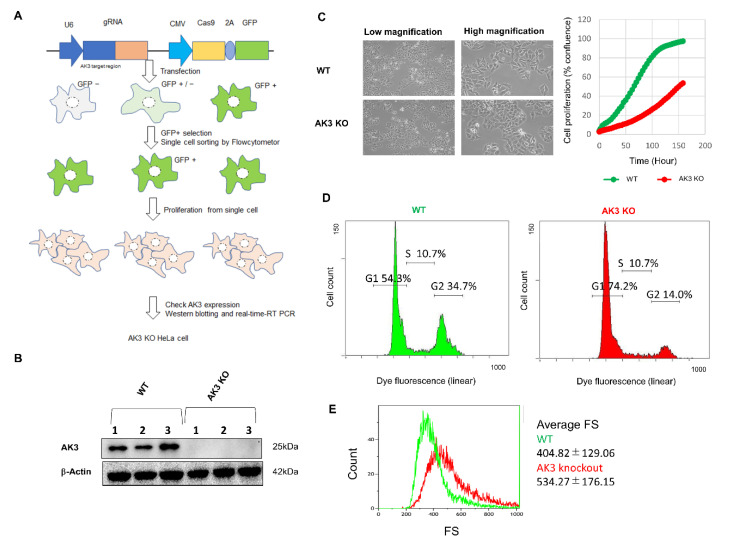
Changes in adenylate kinase 3 knockout (AK3KO) cell. (**A**) Schematic diagram of AK3KO cell production. Plasmid with AK3-targeted genomic RNA (gRNA) exposed to U6 promoter and clustered regularly interspaced short palindromic repeats (CRISPR)-associated protein 9 (Cas9) and green fluorescent protein (GFP) with cytomegalovirus (CMV) promoter was introduced into HeLa cells. Then, GFP co-expressing cells were single-sorted using flow cytometry. Cells grown from a single cell were regarded as AK3KO HeLa cells. Then, each clone was cultured and AK3 protein expression was checking with Western blotting. The clones which showed no protein and mRNA signals were considered as AK3 KO clones. (**B**) Confirmation of AK3KO cell lines from different clones using Western blotting. (**C**) Evaluation of cell morphology (left) and proliferation rate (right) and (**D**) cell cycle and (**E**) size using flow cytometry. FS: forward scatter.

To generate AK3KO HeLa cells, a clustered regularly interspaced short palindromic repeats (CRISPR)-associated protein 9 (Cas9) system containing gRNA and a Cas9 expression unit for AK3 and green fluorescent protein (GFP) in a single vector was used. Cells in which Sigma CRISPR is introduced and in which double-strand breaks are caused by Cas9 are efficiently obtained from cells exhibiting the transient luminescence of GFP; therefore, KO cells were created using single cell-sorting for strongly GFP-positive cells with a cell sorter (Figure 1A). 

The absence of AK3 protein expression in the AK3KO cells (Figure 1B) was confirmed and then a comparison of the proliferation of the wild-type (WT) and AK3KO cells was performed, which showed a decrease in AK3KO (Figure 1C, left). Compared to the WT, AK3KO cells had a slightly rounder morphology and tended to adhere to each other (Figure 1C, right). In addition, an analysis of the cell cycle in control (WT) and AK3KO cells using flow cytometry showed that there were fewer AK3KO cells in the G2 phase than there were in the G1 phase (Figure 1D). The size comparison showed that AK3KO cells were larger than the control cells (Figure 1E).

### 3.2. Metabolome Analysis Showed Decrease in ATP and Increase in Phosphoenolpyruvate (PEP) Levels in AK3KO Cells

**Figure 2:**
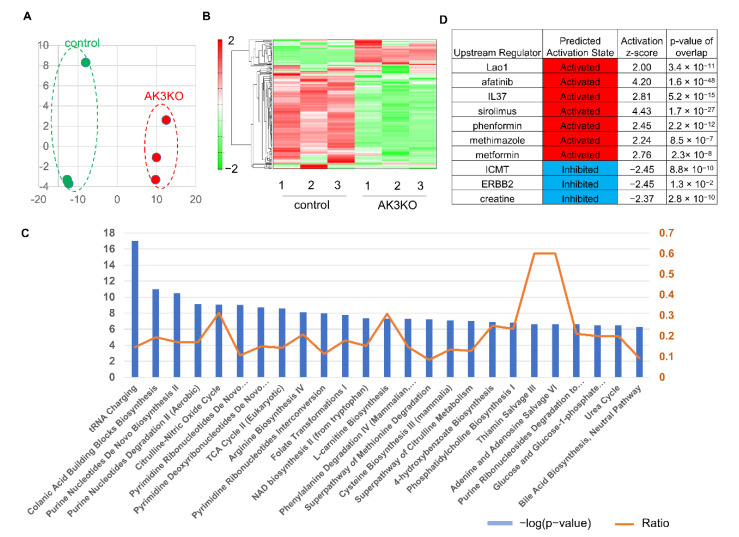
Comprehensive analysis using ingenuity pathways analysis (IPA). (**A**) Principal component analysis (PCA) of normalized metabolic data. Percentage values on axes represent contribution rate of first and second principal components (PC1 and PC2, respectively) to total variation. (**B**) Heat map of hierarchical cluster analysis. Columns indicate WT and AK3KO groups. Rows indicate normalized levels of each metabolite. Dendrogram of each heat map shows relationship to normalized metabolite level patterns. (**C**) Ranking of known pathways using IPA; *p*-values calculated using IPA software are displayed as inverse values. Orange line indicates proportion of genes included in each pathway. (**D**) Upstream regulators identified using IPA; expected activators and inhibitors are shown in red and blue, respectively.

AK3KO was expected to cause changes in metabolites because of potentially unsuccessful GTP + AMP ⇌ GDP + ADP reactions in the mitochondria and, therefore, metabolic changes were evaluated using a metabolomic analysis. The principal component analysis (PCA) showed a clear distinction between WT and AK3KO cells (Figure 2A), and hierarchical clustering showed a clear difference in the abundance of metabolites in WT and AK3KO cells (Figure 2B). The ingenuity pathways analysis (IPA) of altered metabolites identified changes in metabolites related to tRNA charging (aminoacylation), which is involved in protein synthesis as a canonical pathway. 

Changes were also observed in metabolites involved in purine and pyrimidine metabolism, and in the pathways associated with the tricarboxylic acid (TCA) cycle (Figure 2C). An upstream regulator analysis revealed that L-amino acid oxidase (Lao1), sirolimus which is involved in energy metabolism, and metabolism were regulated upstream when phenformin and metformin were activated, whereas creatine production was inhibited (Figure 2D). 

**Figure 3:**
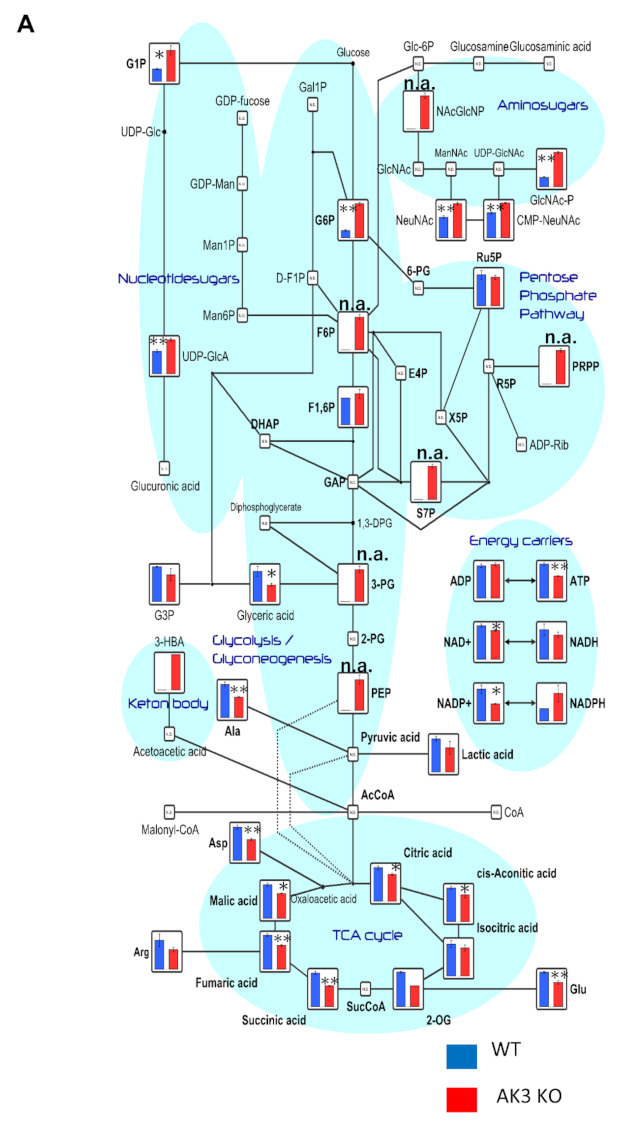

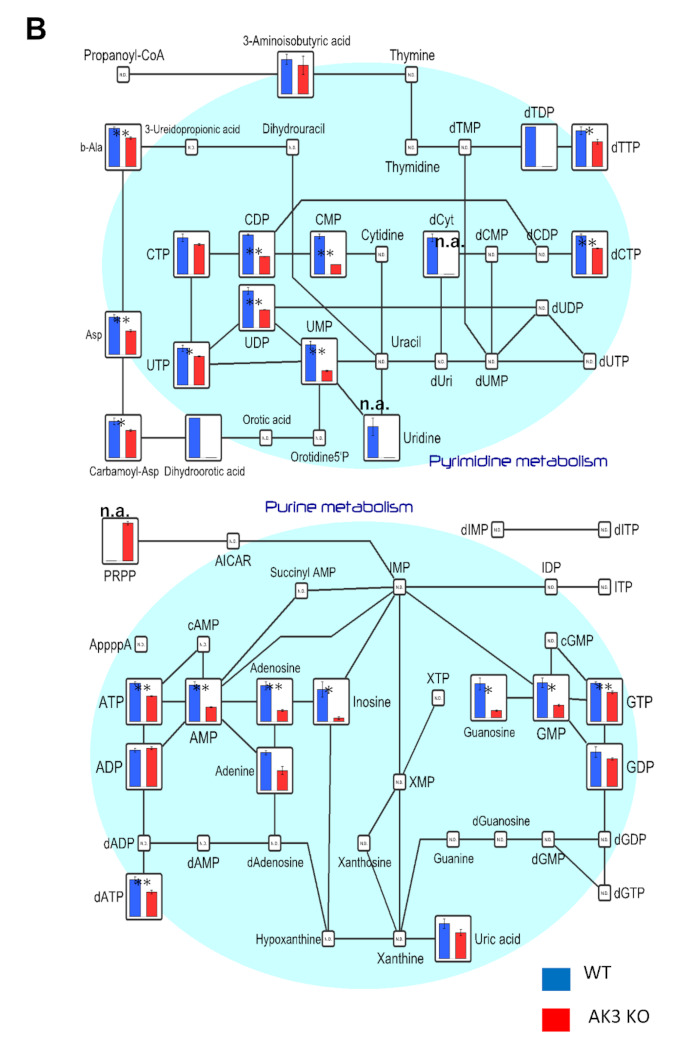
Changes in various metabolites shown using metabolomic analysis. (**A**) Changes in metabolites in glycolysis and the tricarboxylic acid (TCA) cycle. (**B**) Changes in metabolites associated with pyrimidine and purine. * *p* < 0.05, ** *p* < 0.01.

In addition, AK3KO cells showed an increase in glycolytic metabolites other than lactic acid, such as glucose 6 phosphate (G6P), fructose 6 phosphate (F6P), and phosphoenolpyruvate (PEP), whereas most TCA cycle metabolites tended to decrease in the AK3KO cells. In addition, the PRPP of the pentose phosphate pathway also increased (Figure 3A). Among the metabolites involved in nucleic acid metabolism, we noted a decrease in ATP and decrease in guanine nucleotides such as GTP (Figure 3B).

### 3.3. Serial Analysis of Gene Expression (SAGE) Showed AK3KO Changed Expression of Genes Related to Oxidative Stress

**Figure 4:**
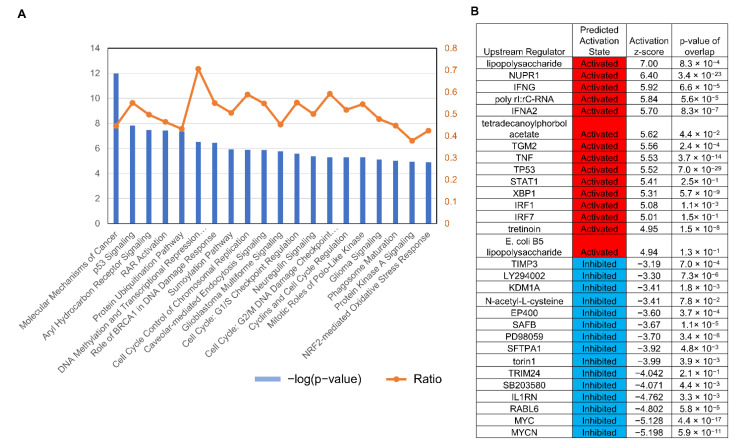
Evaluation of gene expression using serial analysis of gene expression (SAGE) analysis. (**A**) Selected significantly enriched canonical pathways identified using ingenuity pathway analysis (IPA). Diagram shows significantly overrepresented canonical pathways. Multiple-testing corrected *p*-value was calculated using Benjamini–Hochberg method to control rate of false discoveries in statistical hypothesis testing. Ratio represents number of molecules in given pathway that meet cut-off criteria, divided by total number of molecules that belong to the function. Brown, blue, and grey bars indicate positive z-score, negative z-score, and no available activity pattern, respectively. (**B**) List of top 15 and bottom 15 genes with major expression changes; red and blue show increased and decreased expression, respectively.

A microarray analysis investigating the changes in gene expression in AK3KO HeLa cells showed alterations in genes related to activities such as cell proliferation and the oxidative stress response, identified as canonical pathways (Figure 4A). The activation of the upstream regulator NUPR1, which is a stress protein, and XBP1, which is related to endoplasmic reticulum (ER) stress, as well as the suppression of MYC were observed (Figure 4B). Expression levels of the AK isozymes AK4, AK5, and AK9 were higher in AK3KO HeLa cells than they were in control HeLa cells. Similar to AK, we noted a decrease in the expression of creatine kinase B (CKB), an enzyme involved in ATP energy metabolism, whereas the expression of p21, which is related to the cell cycle, increased. Among the antioxidant enzymes, the expression of mitochondrial superoxide dismutase 2 (SOD2) and SOD3 present in the extracellular space was observed.

### 3.4. Intracellular ATP Level Decreased and Mitochondrial DNA Increased in AK3KO Cells

**Figure 5:**
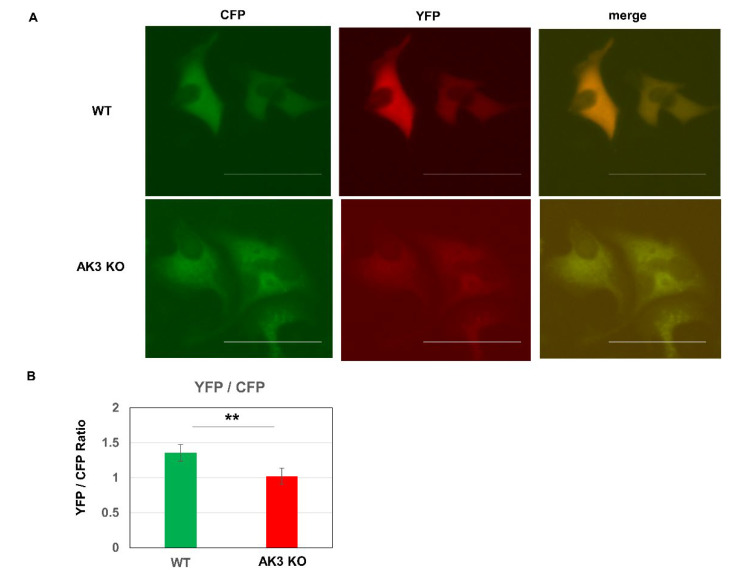
Analysis of proliferation and energy metabolism of adenylate kinase 3 knockout (AK3KO) cells. (**A**) Evaluation of intracellular ATP: Ateam plasmid was introduced to evaluate intracellular ATP levels and its expression was analyzed. Fluorescence of cyan fluorescence protein (CFP, 475 nm) and yellow fluorescence protein (YFP (527 nm) were evaluated for ATP non-binding and ATP-binding, respectively. (**B**) Comparison of YFP/CFP expression. ** *p* < 0.01.

Evaluation of the intracellular ATP level using the fluorescence resonance energy transfer (FRET)-based fluorescent ATP probe “ATeam,” showed lower whole cell levels in AK3KO cells than in WT cells (Figure 5A,B). 

### 3.5. Phosphoenolpyruvate Carboxykinase 2 (PCK2) Complements AKO3KO-Induced GDP Deficiency 

**Figure 6:**
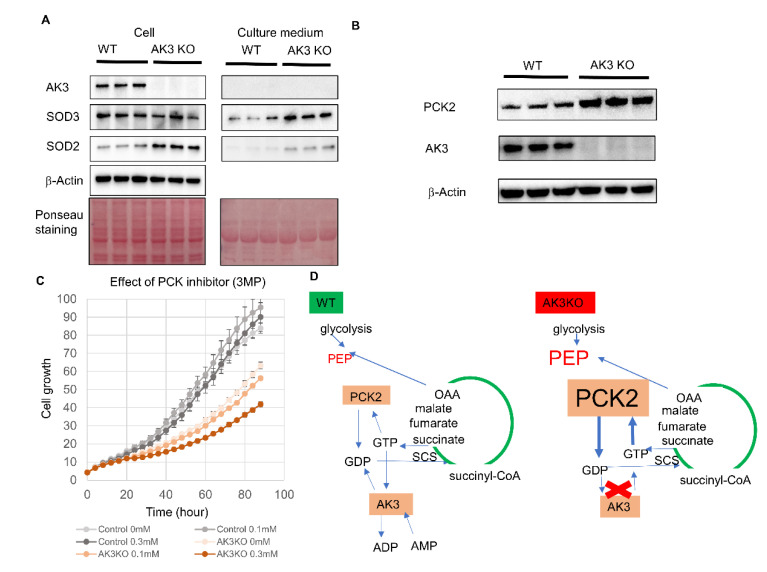
Evaluation of changes in protein expression in adenylate kinase 3 knockout (AK3KO) cells and effect of phosphoenolpyruvate carboxykinase 2 (PCK2) inhibition on AK3KO HeLa cell proliferation. (**A**) Evaluation of intracellular and extracellular protein expression using Western blotting. (**B**) Expression of PCK2 protein in AK3KO cells. (**C**) Effect of PCK2 inhibitor 3MP on proliferation of AK3KO and control HeLa cells. (**D**) Functions of PCK2 and AK3 in wild-type HeLa (HeLaWT) (left) and AK3KO (right) cells. In WT, AK3 and PCK2 produce GDP from GTP, but in AK3KO, PCK2 expression increases to complement reaction producing GDP from GTP.

The results of the suppression of ATP production in AK3KO and the increased mRNA expression of SOD2 and SOD3 suggested that oxidative stress was enhanced. We evaluated SOD2 and SOD3 protein expression using Western blotting (WB). It is likely that SOD2 levels increased intracellularly and extracellularly, whereas SOD3 only increased extracellularly in AK3KO HeLa cells. Furthermore, AK3KO increased oxidative stress and enhanced SOD protein expression, which eliminated oxidative stress (Figure 6A). Metabolomic and microarray analyses identified an increase in PEP and in mitochondrial PCK2, respectively levels in AK3KO HeLa cells. Therefore, WB was used to determine any potential changes in the protein levels, which showed an increased expression of PCK2 (Figure 6B). Increased PCK expression was considered to be involved in the proliferation of AK3KO cells and, therefore, its effect as evaluated using 3-mercaptopicolinic acid (MPA), which is an inhibitor of PCK. MPA showed no effect on the proliferation of control HeLa cells at up to 0.3 μM, whereas the growth of AK3KO HeLa cells was suppressed at concentrations from 0.1 μM, suggesting that PCK was required for AK3KO HeLa cell growth (Figure 6C). 

## 4. Discussion

AK3 supplies the GDP necessary for the TCA cycle reaction in the mitochondrial matrix that converts succinyl-CoA to succinic acid. Consequently, we predicted that knocking out AK3 would inhibit the TCA cycle due to an imbalance between GDP and GTP in the mitochondria and, therefore, would reduce the ATP production capacity in the mitochondria. A metabolomic analysis showed a decrease in ATP and guanine nucleotides, but not to the extent expected and, thus, we considered that a complementary factor was likely to be in operation. PEP is produced in glycolysis and metabolized to pyruvate, which is converted to acetyl-CoA by pyruvate dehydrogenase and then it enters the TCA cycle, after which GTP is produced by succinyl-CoA synthetase. PCK2 (PEPCK-mitochondria type) consumes oxaloacetate and GTP to produce PEP and GDP, and the process of transporting PEP from the mitochondrial matrix to the cytoplasm is known as the PEP cycle [24]. Furthermore, mitochondrial GTP has been reported to be important for nutrient sensing, mitochondrial maintenance, and pancreatic β-cell health [25]. The metabolomic analysis showed an increase in PEP, and Western blotting also showed an increase in PCK2 expression, which is related to the increase in PEP. Therefore, we believe that PCK2 expression was increased to supply GDP to compensate for GTP deficiency. Furthermore, these results suggests that the expression of PCK2, which catalyzes the action producing GDP from GTP, is enhanced to complement the GDP deficiency induced by AK3KO in HeLa cells. In addition, while the PCK2 inhibitor MPA did not inhibit the growth of control HeLa cells, it inhibited that of the AK3KO HeLa cells, which supports the notion that GDP from the PCK2 reaction is necessary for growth. In addition to PCK, nucleoside diphosphate kinase (NME) is an enzyme involved in the reaction that produces GDP and, in particular, NME4 is localized in the mitochondrial matrix [26]. Consequently, we expected the NME expression to increase in a compensatory manner, but the microarray analysis did not show any significant changes.

The microarray analysis revealed changes in nuclear factor-erythroid factor 2-related factor 2 (Nrf2)-mediated oxidative stress gene expression involved in oxidative stress. Furthermore, we detected an altered expression of SOD2 and SOD3, which are antioxidant enzymes localized in the mitochondrial matrix and extracellular space, respectively. The expression of SOD3 decreases as cancer progresses, and the non-involvement of SOD3 is considered to enhance the growth of cancer. Furthermore, SOD3 expression is known to impart resistance to oxidative stress and suppress metastasis [27]. 

The previous study reported that the maintenance of high SOD3 expression in cancer cells is important for suppressing cancer progression [28]. Hence, the enhanced expression of SOD3 resulting from knocking out AK3 is interesting as a potential factor in suppressing cancer. Increased expression of antioxidant enzyme genes and proteins is thought to be due to metabolic abnormalities caused by AK3KO, resulting in mitochondrial dysfunction with increased cellular oxidative stress. 

Both AK3 and AK4 are localized in the mitochondrial matrix, and we believe that the increased AK4 expression in this study was likely stress-induced and mediated by the increase in oxidative stress caused by AK3KO. Substrate competition may likely exist between AK3 and AK4 and, therefore, a detailed analysis of the relationship between these AK isozymes would be necessary in future studies.

In conclusion, the findings of this study suggest that mitochondrial dysfunction caused by abnormal nucleotide metabolism mediated by AK3KO may be involved in the oxidative stress response. Our findings also show that changes in the quantitative relationship between the metabolites GDP and GTP due to abnormal nucleotide metabolism may be related to proliferation. Finally, we anticipate that the effect of AK3 on metabolic abnormalities will be elucidated in a future detailed analysis of oxidative stress, proteins involved in the conversion reaction between GDP and GTP, and actual metabolites in AK3KO cells.

## 5. Conclusions

AK3KO decreased cell proliferation and increased the oxidative stress-related proteins, SOD2 and SOD3, suggesting the importance of this enzyme in maintaining mitochondrial homeostasis. The complementary upregulation of PCK2 to increase GDP and address the deficiency caused by AK3KO further indicated the importance of AK3 in GDP synthesis in the mitochondrial matrix.

## Data Availability

Not applicable.
